# Supplementary material for: Development and Validation of a 54K Genome-Wide Liquid SNP Chip Panel by Target Sequencing for Dairy Goat
Source: Genes (Basel). 2023 May 22;14(5):1122. doi: 10.3390/genes14051122 (PMC10218471; doi:10.3390/genes14051122)
Supplement: Supplementary file 1 [file genes-14-01122-s001.zip › Supplementary_Methods.pdf]

Supplementary Methods

Selective signatures associated with phenotypic traits based on SNPs.

We compared the 133 commonly selected regions associated with different phenotypes (e.g., production, morphological and physiological traits) by XP-CLR, π-ratio and iHS analyses as well as the 95 commonly selected regions by XP-CLR, π-ratio and HKA analyses with previously mapped QTLs. We found that 30 of the selective sweeps by XP-CLR, π-ratio and iHS and 43 of the selective sweeps by XP-CLR, π-ratio and HKA overlapped with known QTLs69, which are associated with a number of traits such as such as reproduction, milk, growth, meat and carcass, fat deposition in the tails and wool fineness (Supplementary Table S5, Supplementary Data S43 and S44). Specifically, for XP-CLR, π-ratio and iHS, the number of QTLs (shorter than 100 Mb) overlapping for traits such as reproduction, milk, growth, meat and carcass, fat deposition in the tails are 3 (permutation test, $P < 0.01$), 1 (permutation test, $P < 0.05$), 20 (permutation test, $P < 0.001$), 7 (permutation test, $P < 0.05$), respectively (Supplementary Table S5 and Supplementary Data S43). For XP-CLR, π-ratio and HKA, the overlapping numbers of QTLs (shorter than 100 Mb) for the above traits are 1 (permutation test, $P < 0.001$), 4 (permutation test, $P < 0.001$), 32 (permutation test, $P < 0.001$) and 2 (permutation test, $P < 0.05$), respectively (Supplementary Table S5 and Supplementary Data S44).
